# DsbA is a redox-switchable mechanical chaperone†

**DOI:** 10.1039/d1sc03048e

**Published:** 2021-07-19

**Authors:** Edward C. Eckels, Deep Chaudhuri, Soham Chakraborty, Daniel J. Echelman, Shubhasis Haldar

**Affiliations:** Department of Biological Sciences, Ashoka University Sonepat Haryana 131029 India shubhasis.haldar@ashoka.edu.in; Department of Biological Sciences, Columbia University New York NY 10027 USA ece7001@nyp.org; Department of Internal Medicine, Columbia University Medical Center New York NY 10032 USA

## Abstract

DsbA is a ubiquitous bacterial oxidoreductase that associates with substrates during and after translocation, yet its involvement in protein folding and translocation remains an open question. Here we demonstrate a redox-controlled chaperone activity of DsbA, on both cysteine-containing and cysteine-free substrates, using magnetic tweezers-based single molecule force spectroscopy that enables independent measurements of oxidoreductase activity and chaperone behavior. Interestingly we found that this chaperone activity is tuned by the oxidation state of DsbA; oxidized DsbA is a strong promoter of folding, but the effect is weakened by the reduction of the catalytic CXXC motif. We further localize the chaperone binding site of DsbA using a seven-residue peptide which effectively blocks the chaperone activity. We found that the DsbA assisted folding of proteins in the periplasm generates enough mechanical work to decrease the ATP consumption needed for periplasmic translocation by up to 33%.

## Abstract 2

Protein translocation is facilitated by DsbA chaperone in a redox-dependent manner.

## Graphical abstract

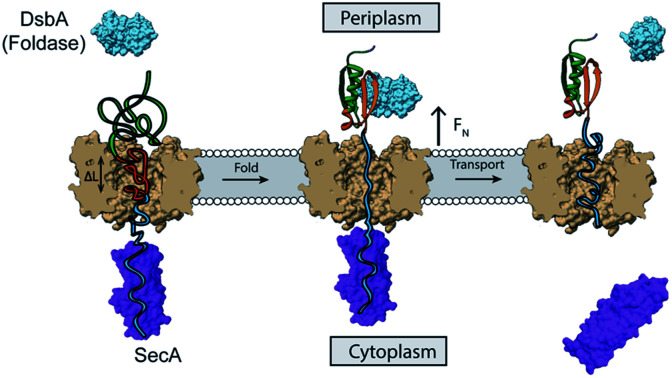

## Introduction

Peptide translocation is of utmost importance in bacteria, where a large number of proteins, including virulence factors, must pass through the translocon pore in the unfolded state and properly fold into the native configuration in the periplasm.^1–5^ A large number of proteins secreted into the periplasmic space contain disulfide bonds which are introduced by the oxidoreductase enzymes of the Dsb family, which share a conserved thioredoxin-type fold.^6,7^ The prototypical family member, DsbA, is necessary for the maturation of a range of virulence factors including flagellar motors and pilus adhesins, type III secretion systems, and heat-labile and heat-stable enterotoxins and has emerged as a target for novel antibiotic development.^8–11^ Studies showed that DsbA assists the efficient secretion of cysteine-null proteins, indicating its ability to engage unfolded substrates during translocation.^14^

The well-studied eukaryotic chaperones BiP and Hsp70 are known to facilitate the translocation of peptides into the endoplasmic reticulum and mitochondria, respectively.^15–17^ It is believed that chaperone binding to an unfolded substrate protein on one side of the membrane can induce biased transport of a polypeptide through a Brownian ratchet mechanism.^18–21^ This relies not on the energy of hydrolysis of ATP but on the differential concentrations of the chaperones on either side of the membrane. Simple binding of chaperones on one side of the membrane prevents the backsliding of the protein into the pore, facilitating the directional transport of the polypeptide. Once the polypeptide is properly folded on the other side of the membrane, it is too large to pass back through the pore. Although DsbA is considered to be a ‘weak’ chaperone, the effect of oxidation on its chaperone activity or periplasmic translocation is not known.^12,13,66^ If DsbA can indeed act as a redox-dependent chaperone under the physiological constrain, faced by the protein in the translocon pore, it may play a much broader role in periplasmic secretion than previously recognized.

Peptides emerging from nanoscale tunnels such as the translocon pore are known to experience an effective stretching force due to molecular confinement.^22–24^ As such, these peptides emerge from the pore in a linear, extended state and can either collapse on the mouth of the pore or engage with soluble chaperones in the periplasm. Here we apply single molecule magnetic tweezers assay to mimic the entropic stretching forces of the translocon pore. Using this assay, soluble DsbA is shown to cleave and reform disulfide bonds in a model immunoglobulin protein domain, validating the chaperone activity of the oxidoreductase enzyme. We further investigate the redox dependent chaperone activity of DsbA with cysteine free substrates using the globular B1 domain of protein L, which undergoes an equilibrium between the folded and unfolded states under small pulling forces (4–9 pN). We find that the presence of soluble DsbA in the experimental buffer greatly increases the residence time of protein L in the folded state and allows the protein L domain to refold at higher forces. Using the residence time of protein L in the folded state as our metric, we demonstrate the ability to turn DsbA chaperone activity “on” and “off” by changing the redox state of its active site disulfide bond or by targeting its putative binding site with a small inhibitory peptide. These results suggest that DsbA acts as a mechanical foldase that can engage and fold peptides under an effective stretching force, such as those emerging from the translocon pore.^54,67^ Because DsbA drives the folding of its substrates at higher forces, it increases the amount of mechanical work performed by the folding substrate, which in turn decreases the energy needed to liberate it from the pore. We calculate that the work done by chaperone-assisted protein folding on the periplasmic side of the translocon pore could supply one-third of the energy required for the protein L translocation, lowering the ATP consumption to only 2 molecules per protein L polypeptide translocated through the SecA motor.

## Results

### Monitoring single molecule oxidative folding by DsbA

**Fig. 1:**
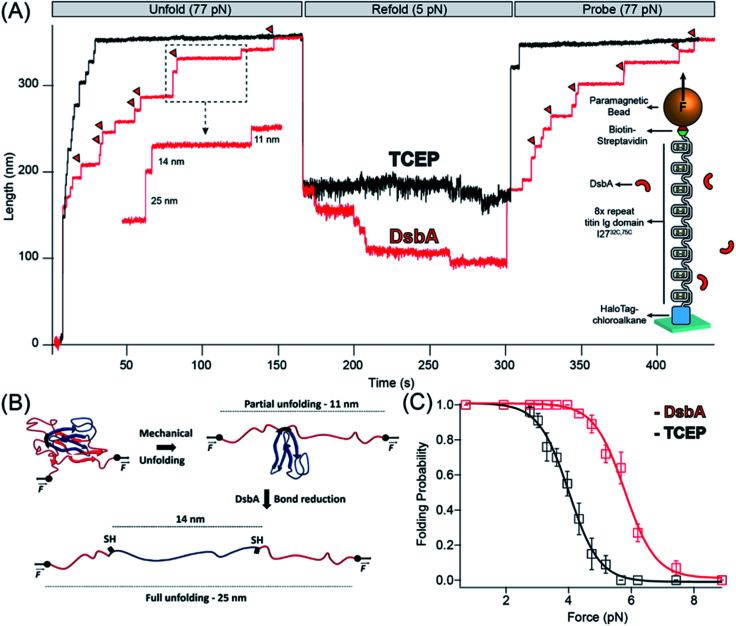
Chaperone activity of DsbA monitored in disulfide bonded protein: (A) reduction of I27 by TCEP and DsbA: in the presence of reducing conditions maintained by 10 mM TCEP (black trace), the polyprotein is unfolded at a denature pulse of 77 pN, causing a mixture of six 11 nm (due to mechanical unfolding) and 14 nm (due to disulfide reduction) steps along with two already reduced 25 nm steps. After unfolding, the force is quenched to 5.2 pN for the observation of refolding events. A final probe pulse at 77 pN reveals only a single 25 nm step, representing the refolding of a single I27 domain in its reduced state. With DsbA (red trace), unfolding at a denature pulse at 77 pN results in similar seven 11 and 14 nm steps (red triangles indicating enzymatic disulfide reduction) with a single 25 nm step, but after quenching the force to 5.2 pN, seven downward folding steps occurred, in contrast to only one folding step in the presence of TCEP. A final probe pulse shows six mixed steps of 11 and 14 nm and one 25 nm step, indicating the re-oxidation of six Ig domains. (B) Schematics of complete unfolding of a titin Ig domain under force: application of mechanical force on I27^C32–C75^ first unfolds the protein up to the internal disulfide bond, leading to an extension of 11 nm (of the un-sequestered peptide) which is followed by an additional extension of 14 nm due to reduction of the disulfide bond. (C) Folding probability increased in the presence of DsbA: folding probability as a function of force is plotted in the presence of TCEP (black) and DsbA (red). The folding probability is shifted to a higher force in the presence of DsbA, with the force required for 50% refolding probability increasing from 4.1 to 5.8 pN. More than eight individual trajectories are measured and averaged for each force. More than five individual trajectories are measured and averaged for each force. Error bars are the standard errors of mean (s.e.m.).

We recently developed a single molecule assay to study chaperone activity using magnetic tweezers-based force spectroscopy that overcomes the limitations of bulk studies of chaperones.^25–28^ By their nature, bulk studies examine chaperone–client interactions in an environment far from *in vivo*, and denaturants such as guanidine, low pH, or high temperature used in these experiments necessarily perturb the structure of the chaperone as well as the substrate protein. Single molecule magnetic tweezers have the unique advantage of using mechanical force to denature only the substrate protein without disturbing chaperone molecules in the surrounding solution. The mechanical unfolding of individual proteins is achieved through the attachment of the substrate protein to microscopic probes; in our magnetic tweezers-based force spectroscopy, proteins are tethered at their N-terminus to a glass surface *via* HaloTag-Halo ligand covalent chemistry,^27,29^ and at their C-terminus to a paramagnetic bead *via* biotin-streptavidin^30^ (Fig. 1A, inset). The positioning of a permanent magnet at fixed distances from the paramagnetic bead applies a passive force clamp that can explore a broad range of forces up to ∼100 pN. Furthermore, the long-term stability of magnetic tweezers enables the measurement of slow rates, with successive measurements from a single protein achieving a record of two weeks.^27^

We used the magnetic tweezers assay to first measure the oxidoreductase activity of DsbA on a model titin immunoglobulin domain, I27^C32–C75^, containing a single disulfide bond.^31,32^ The recombinant protein construct was engineered with eight tandem repeats of I27^C32–C75^ to provide a molecular fingerprint upon unfolding. The unfolding of an oxidized I27^C32–C75^ domain containing a formed disulfide bond results in an 11 nm extension^25,31^ of the polypeptide chain (Fig. 1A and B). The introduction of tris carboxyethyl phosphine (TCEP) into the solution results in the cleavage of the I27^C32–C75^ disulfide bond and release of a cryptic length of 14 nm from the polypeptide sequestered behind the disulfide bond (Fig. 1A and B). In the assay, the eight tandem repeats are unfolded during the “denature” pulse at a pulling force of 77 pN in the presence of reduced 50 μM DsbA (Fig. 1A, red trace) or 10 mM TCEP (Fig. 1A, black trace). The protein is left at this high force for sufficient time to allow for complete unfolding (both the mechanical unfolding, 11 nm steps and disulfide cleavage 14 nm steps) of all eight I27^C32–C75^ domains in the polyprotein. The force is then “quenched” to 5.2 pN to allow for refolding for ∼150 seconds, followed by a subsequent “probe” pulse back to 77 pN. The probe pulse allows for the counting of the number of re-oxidized I27^C32–C75^ domains, which is measured as a function of the quench force. In the two recordings shown in Fig. 1B, seven I27^C32–C75^ domains refolded during the quench pulse in the presence of DsbA, whereas only one domain refolded in the presence of the reducing agent TCEP. Of the seven domains that refolded in the presence of DsbA, six contained reformed disulfide bonds (11 nm steps), while one refolded with its cysteines still reduced (25 nm step).

This assay was performed for many cycles, and Fig. 1C summarizes the folding probability of I27^C32–C75^ in the presence and absence of DsbA. The folding probability, *P*_f_, describes the likelihood of a single domain to be folded at a particular force (see ESI Fig. 1† and Methods). The half-point force for I27^C32–C75^ (denoted as folding probability = 0.5) shows a prominent rightward shift from 4.1 to 5.8 pN in the presence of DsbA. A similar shift in the folding probability of I27^C32–C75^ was also noted for protein disulfide isomerase (PDI), a eukaryotic oxidoreductase from the thioredoxin family.^25^ The folding step size noted during the quench pulse indicates that folding occurs before the introduction of the disulfide bond,^33,34,66^ suggesting that there is acceleration of the folding process because the chaperone is likely independent from conformational restriction by a newly introduced disulfide bond (ESI Fig. 7†).

### DsbA chaperones a cysteine-free substrate in a redox dependent manner

**Fig. 2:**
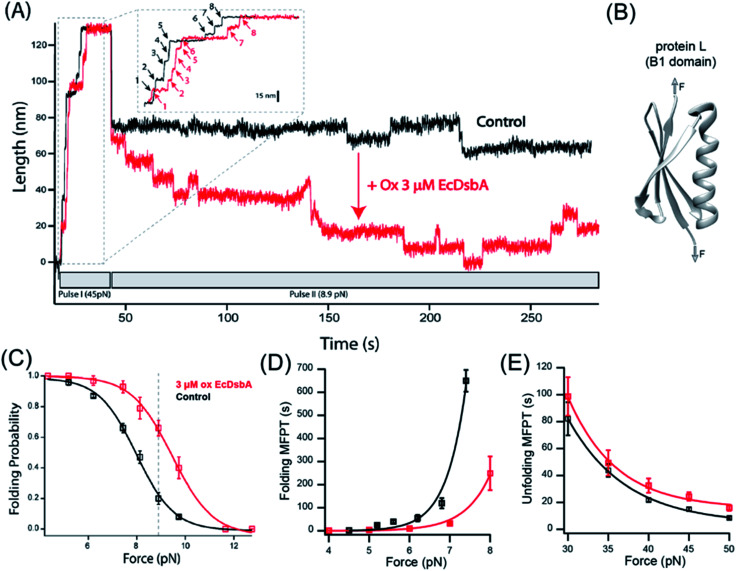
DsbA act as a chaperone for the protein L domain: (A) magnetic tweezers force-clamp trajectory of a protein L octamer in the presence (red) and absence (black) of 3 μM oxidized DsbA at 8.9 pN. In pulse I, the protein is unfolded at 45 pN, where the 8 protein L unfolding events are observed as steps of ∼15 nm (see the inset for the horizontally magnified trace). In pulse II, force is quenched to 8.9 pN, where an initial relaxation refolding is observed as downward steps of ∼9.5 nm, followed by equilibrium unfolding/refolding steps. (B) B1 domain of protein L showing a simple α/β fold without any cysteine or disulfide moieties. (C) Folding probability as a function of force in the presence (red) and absence (black) of DsbA. The rightward shift of the curve in the presence of DsbA indicates the mechanical chaperone activity of DsbA that enhances the folding of protein L in the force range from 4–11 pN. The maximum difference in the folding probabilities, marked by the gray line, occurs at 8.9 pN, with the force demonstrated in panel (A). Error bars represent the standard errors of the mean of ≥5 independent molecules. (D) Variation in the MFPT of refolding: the MFPT of refolding is plotted as a function of force in the absence (black) and presence (red) of DsbA. The MFPT data were fit with a single exponential equation MFPT(*F*) = *A*e^−*F*/*φ*^. More than eight individual trajectories are measured and averaged for each force. Error bars are the standard errors of mean. (E) Variation in the MFPT of unfolding: DsbA negligibly delays the unfolding kinetics by marginally increasing the time of complete unfolding of all eight domains. The difference in the MFPT at the lowest unfolding forces (30 and 35 pN) is not statistically significant. More than eight individual trajectories and more than sixty unfolding events have been calculated for each force. Error bars are the standard errors of mean (s.e.m.).

The B1 antibody light chain binding domain of protein L (hereafter referred to as “protein L”) from *Finegoldia magna* is a model substrate well described in bulk biophysical and single-molecule force spectroscopy techniques.^35–38^ Protein L is 62 residues in length with a simple α/β fold (Fig. 2B) that, importantly, lacks any metal cofactors, or cysteine or proline residues.^39^ In all studies herein, we employ an 8-repeat tandem modular protein L, flanked with N-terminal HaloTag and C-terminal biotin for tethering in the same geometry as that used for I27^C32–C75^. With the application of a high mechanical force (45 pN in ESI Fig. 1A, pulse I†), the eight protein L repeats unfold as eight discrete stepwise extensions of 15 nm, providing the distinct single-molecule fingerprint of the polyprotein (Fig. 2A, inset). Upon a decrease in force, the total length of the fully unfolded polypeptide collapses due to polymer entropy, followed by discrete stepwise contractions from individual protein L domain refolding (9.0 nm; ESI Fig. 1A, pulse II†). After several seconds, an equilibrium is reached between the stepwise extensions of unfolding and the stepwise contractions of refolding, with an identical length for each transition (ESI Fig. 1A, pulse II†). The equilibrium behavior is reported by the folding probability *P*_f_ as described previously^26^ (ESI Fig. 1A,† and also Methods).

For protein L, the equilibrium position between folding and unfolding is sharply force-dependent (Fig. 2C), with the folding probability shifting from >99% at 4 pN to <1% at 12 pN. In the absence of DsbA, the 50% folding probability is found at 8.0 pN. Upon addition of 3 μM oxidized DsbA to the magnetic tweezers experiment, a shift in the folding probability is observed (Fig. 2A). At an equilibrium force of 8.9 pN, the protein L polyprotein transitions between its 5th, 6th, and 7th folded states in the presence of oxidized DsbA (Fig. 2A, red trace), whereas in the absence of oxidized DsbA the polyprotein transitions between the fully unfolded and the 1st folded state (Fig. 2A, black trace). At the extremes of 4 pN and 12 pN, folding is unaffected, with either 100% of the substrate folded (at 4 pN) or unfolded (at 12 pN), independent of the presence of the enzyme. However, at intermediate forces, the effect of DsbA becomes more pronounced (Fig. 2C). The largest shift is observed at 8.9 pN (dotted line, Fig. 2C), where the protein L substrate has a 0.66 ± 0.05 probability of being folded when in the presence of oxidized DsbA *versus* a 0.20 ± 0.04 probability of being folded in its absence.

### Effect of DsbA on the mean first passage time (MFPT) of unfolding and refolding of protein L

We systematically explored the effect of DsbA by comparing the kinetics of protein L in both high and low force regimes, with and without 3 μM oxidized DsbA. The kinetic analysis allows one to determine if the shift in the folding probability by DsbA is caused by a decrease of the energy of the folding transition state, an increase in the energy of the unfolding transition state, or both. The kinetics of unfolding and refolding can be characterized by the mean first passage time (MFPT), where the first passage time (FPT) is denoted as the minimum time taken to complete unfolding and refolding of all eight domains. The mean first passage time (MFPT) is determined by averaging FPT over several trajectories.^26^Fig. 2D and E illustrate the comparison of the MFPT of refolding and unfolding in the absence (black) and presence (red) of DsbA. In the presence of DsbA, the MFPT of refolding decreases at a particular force magnitude and thus shows accelerated folding kinetics by DsbA (Fig. 2D). In contrast to refolding, DsbA marginally delays the unfolding kinetics (Fig. 2E) at forces from 30–50 pN. Although the difference is small, it suggests that DsbA may be able to associate with the collapsed protein L substrate as well as the denatured form. Fit parameters for the single exponentials can be found in the ESI.†

### Oxidation dependent activity of DsbA

**Fig. 3:**
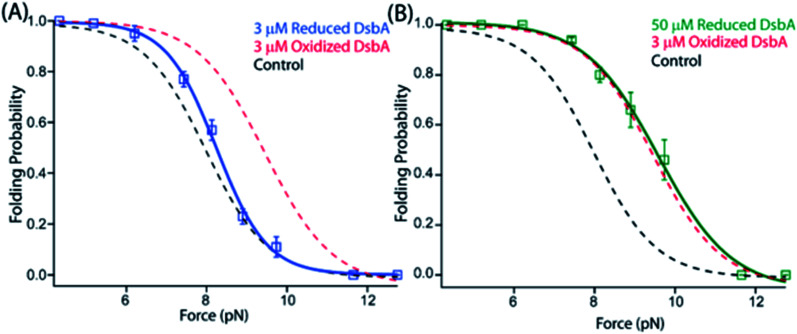
A redox switch controls the chaperone activity of DsbA: (A) the folding probability of 3 μM reduced DsbA (blue squares) has no significant effect on the folding probability of protein L, coinciding with the control experiment (black dotted line). More than five individual trajectories are measured and averaged for each force. Error bars are the standard errors of mean. (B) Increasing the concentration of reduced DsbA to 50 μM restores the mechanical chaperone activity of DsbA. The folding probability of protein L as a function of force in the presence of 50 μM reduced DsbA (green squares) coincides with the folding probability curve of 3 μM oxidized DsbA (red dotted line). The fittings in the absence (black dotted line) and in the presence of 3 μM oxidized DsbA (red dotted line) is plotted as a control. More than seven individual trajectories are measured and averaged for each force. Error bars are the standard errors of mean.

Within the Gram-negative periplasm, oxidized DsbA represents the active fraction that is capable of forming mixed disulfides between the enzyme and substrate peptide. After transferring the disulfide bond into its substrate, DsbA is released with both cysteines of its active site *CXXC* motif reduced. We therefore asked whether the chaperone-like activities of DsbA might depend on its redox state, which is expected if the chaperone activity originates from the hydrophobic groove encapsulating the catalytic site. We reduced freshly purified DsbA with an overnight incubation in 100 μM TCEP (which was then removed with a 10 kDa cutoff concentrating column) and repeated the magnetic tweezers-based mechanical foldase assay. We checked the percentage of the oxidized and reduced DsbA using Ellman's test protocol and described it in detail in the ESI (ESI Fig. 8†). While 3 μM oxidized DsbA shifts the probability of substrate folding to 0.66 at 8.9 pN, 3 μM reduced DsbA (ESI Fig. 2†) induces no such shift in substrate folding (Fig. 3A). Experiments with reduced DsbA tracked the folding probability of the protein L substrate alone over the range of 5–11 pN (Fig. 3A). A comparable foldase effect with reduced DsbA requires 50 μM, a ∼17-fold excess over that of oxidized DsbA (Fig. 3B, green curve and ESI Fig. 3†). Importantly, these experiments with reduced DsbA were performed with 100 μM reducing agent TCEP in solution to prevent spontaneous re-oxidation of the catalytic disulfide bond. These data demonstrate that the alteration of the redox state of the cysteines modulates substrate affinity, possibly by changing the charges or the hydrophobicity of the binding surface, or by altering the overall conformation of the enzyme.^40,41^

### A peptide antagonist blocks the chaperone activities of DsbA

**Fig. 4:**
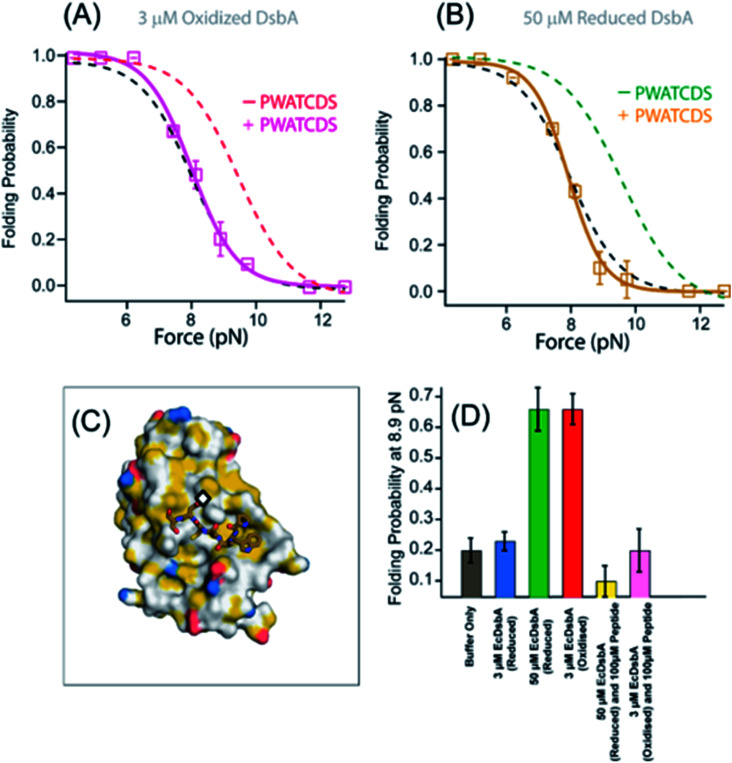
Peptide inhibitor of DsbA maps the chaperone activity to the groove surrounding the catalytic site. (A) The folding probability of protein L in the presence of 3 μM oxidized DsbA and 100 μM PWATCDS peptide (pink squares) is shifted back to the folding probability of the control experiment (no DsbA or peptide, black dotted line), indicating the inhibition of the mechanical chaperone activity of oxidized DsbA (red dotted line fittings). More than five individual trajectories are measured and averaged for each force. Error bars are the standard errors of mean. (B) The mechanical chaperone activity of 50 μM reduced DsbA is also inhibited in the presence of 100 μM peptide (gold squares), aligning with the folding probability in the absence of DsbA (black dotted line fittings). More than five individual trajectories are measured and averaged for each force. Error bars are the standard errors of mean. (C) Structure of *Proteus mirabilis* DsbA with PWATCDS peptide bound. The active site *CXXC* motif is marked with a black diamond. The surface surrounding the active site is largely hydrophobic, as colored according to the schema of Hagemans *et al.* whereby any carbon not bound to a heteroatom is colored yellow. (D) The lower inset shows the folding probability at 8.9 pN in the absence (gray) and presence of 3 μM reduced DsbA (blue), 50 μM reduced DsbA (green), 3 μM oxidized DsbA (red), 50 μM reduced DsbA with 100 μM PWATCDS peptide (yellow) and 3 μM oxidized DsbA with 100 μM PWATCDS peptide (pink).

DsbA has emerged as an attractive therapeutic target due to its vital role in the maturation of bacterial virulence factors, including toxins, adhesins, and secretion machinery.^10,42–44^ For this reason, several peptide inhibitors of DsbA have been developed, based on the oxidoreductase interacting partner DsbB, which binds at and oxidizes the catalytic *CXXC via* interaction by a short loop with the sequence PFATCDF.^8^ Prior studies mutated the PFATCDF sequence at two positions: a point mutation of the N-terminal phenylalanine to tryptophan and a substitution of the N-terminal phenylalanine to serine (to improve the solubility of the peptide).^8^ This produced the peptide PWATCDS with high affinity for the hydrophobic groove of DsbA from *Proteus mirabilis* in a co-crystal structure^9^ (Fig. 4C). The binding of PWATCDS effectively blocks the oxidoreductase activity of DsbA. We therefore asked whether this peptide binding at the hydrophobic groove neighboring the *CXXC* motif would also inhibit the mechanical foldase activity of DsbA.

We repeated the mechanical foldase assay with protein L and either reduced or oxidized DsbA, using magnetic tweezers, now in the presence of the PWATCDS peptide. With 3 μM oxidized DsbA at 8.9 pN, the folding probability of protein L is found to be 0.66; however, with the addition of the peptide at 100 μM, the folding probability shifts to 0.21, suggesting a loss of the enzyme's foldase activity (Fig. 4A, pink curve and ESI Fig. 4†). Furthermore, the inhibitory effect is evident at all forces tested (4–12 pN), as the folding probability of protein L in the presence of oxidized DsbA and 100 μM peptide closely tracks the folding probability of the control experiment.

This indicates that the cysteine residue of this peptide appears to be critical for the mixed disulfide formation and might change oxidation state of the enzyme. To further verify whether it originates from the change in the oxidation state or binding to the hydrophobic groove, we performed an experiment with reduced DsbA in the presence of the peptide. We observed that the residual foldase activity of 50 μM reduced DsbA is also lost upon addition of 100 μM PWATCDS peptide (Fig. 4B, gold curve and ESI Fig. 5†). This is certainly attributed to the binding of the peptide to the hydrophobic groove, surrounding the catalytic site of the enzyme, which can also be confirmed from the crystal structure of the DsbA-PWATCDS complex.^9^ A comparison at a highly sensitive equilibrium force of 8.9 pN demonstrates large shifts in the folding probability of protein L with or without the catalytic thiols oxidized *versus* in the presence or absence of the PWATCDS peptide (Fig. 4D). Notably, the peptide alone does not affect the folding of protein L (ESI Fig. 9†).

### Equilibrium folding energy calculation of protein L in the presence and absence of DsbA

The equilibrium folding energy of protein L has been determined as a function of force, based on Boltzmann distribution, using the following equation, as described by Chen *et al.*^65^
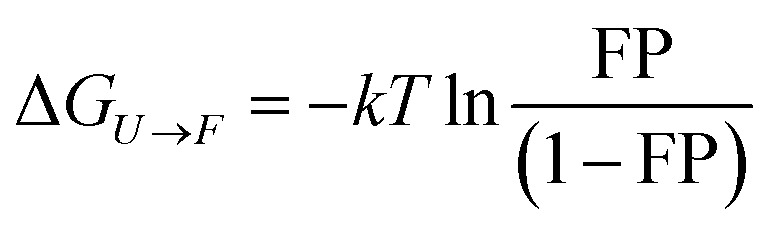


ESI Fig. 6† shows the comparative data of the free energy difference (Δ*G*) within a span of 6–10 pN force, where both folding–unfolding transitions occur within the experimental time-scale. Our result shows that, at a given force, the free energy of folding of protein L, in the presence of oxidized DsbA, is much less than that in the absence of DsbA (ESI Fig. 6A†). This clearly shows the foldase nature of oxidized DsbA. However, in the presence of reduced DsbA, at 3 μM, the free energy of folding is almost the same as that of the control. But, in the presence of 50 μM reduced DsbA, the free energy of folding decreases, which indicates that the energy landscape shifted towards to a folded state (ESI Fig. 6B†). The addition of peptides to 3 μM oxidized or 50 μM reduced DsbA decreases the folding as in both the cases the free energy of folding is higher in comparison to that without peptides (ESI Fig. 6C and D†).

## Discussion

By applying single-molecule force spectroscopy using a novel magnetic tweezers assay, we have provided an empiric description and measurement of a redox-driven chaperone behavior of *E. coli* DsbA. We use a protein domain that lacks cysteines to show that the chaperone activity of DsbA is separable from its oxidoreductase activity. Based on our observations, we attribute the acceleration of folding to transient noncovalent interactions between DsbA and its substrate that do not involve thiol/disulfide exchange with the said substrate. We have further been able to localize this interface to the hydrophobic groove surrounding the *CXXC* catalytic motif by taking advantage of the groove affinity for its DsbB electron shuttling partner. Reducing the catalytic thiols or binding of the PWATCDS peptide both alter the interface between DsbA and the substrate protein L such that the chaperone activity is weakened or abrogated. Determining whether these two modifications of the DsbA enzyme alter electrostatics, hydrophobicity, or local geometry would help to elucidate the mechanism by which DsbA recognizes and folds its protein L substrate.^45^ Interestingly, the conformational changes of DsbA upon oxidation/reduction of its disulfide bond have been noted previously,^40,41^ and may contribute to the results seen in this study. Although this is the first report of redox-controlled mechanical foldase activity for DsbA, there are several other instances of chaperones with redox activated switches. Such a mechanism can be rationalized as a protective response to increased oxidative stress, which is known to alter side chain chemistry and cause an accumulation of misfolded proteins. Peroxiredoxins act as oxidoreductases in a monomeric form but oligomerize into molecular chaperones under the conditions of oxidative stress.^46,47^*S*-Nitrosylation, a by-product of reactive nitrogen stress, at a non-catalytic thiol of human thioredoxin regulates its redox and anti-apoptotic activity.^48^

**Fig. 5:**
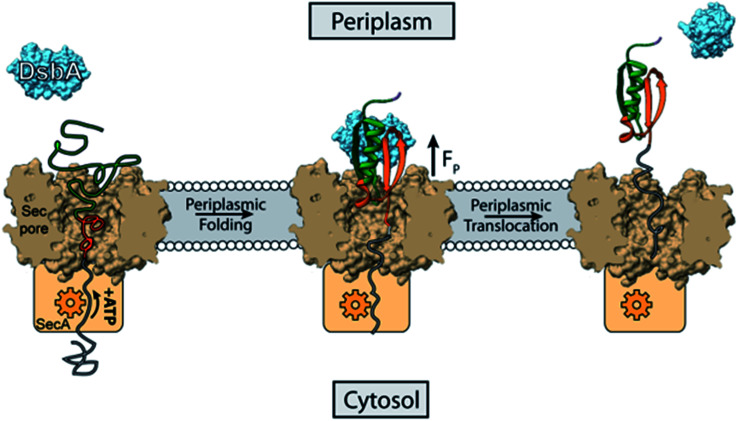
DsbA accelerated transport across the translocon pore. Translocation of peptides into the periplasm takes place with the help of Sec machinery, where SecA (orange) translocates the peptide with the help of ATP hydrolysis. Here we show that the portion of the translocated polypeptide that is exposed to the periplasm (green polymer) may interact with oxidized DsbA (light blue) and fold on the mouth of the translocon pore, generating a pulling force (*F*_P_) of several piconewtons. The pulling force strains the remaining polypeptide in the translocon pore (grey polypeptide), overcoming the friction of the tunnel, and pulls it through to the periplasmic side. The work of protein folding therefore reduces the number of rounds of ATP hydrolysis that the SecA motor must undergo to push the polypeptide through the translocon pore.

The foldase activity of DsbA could have broader implications for periplasmic protein quality control. DsbA has been shown, for example, to bind to translocating polypeptides in the periplasm, both co-translationally and post-translationally.^1,49^ Here we propose an alternative version of chaperone biased polypeptide translocation based on our experimentally observed results that could be important for post-translationally secreted polypeptides (Fig. 5). We suggest that the DsbA enhanced folding of protein domains on the periplasmic mouth of the Sec pore can generate a pulling force that transfers its strain to the polypeptide in the translocon tunnel, and to any portion still in the cytosol. Chaperone assisted folding on the periplasmic side of the membrane would then in turn decrease the energetic costs of protein translocation.

Prior to translocation, a protein is first maintained in the unfolded state by the SecB chaperone which carries the unfolded polypeptide to the SecA motor for the transport of the polypeptide through the SecYEG pore using ATP hydrolysis.^50^ It has been shown for the SecA motor that one round of ATP binding and hydrolysis is responsible for the translocation of 20 amino acid residues through the translocon pore,^51^ so a single protein L domain with 60 residues participating in the fold would require 3 ATP molecules for translocation. Assuming a mechanochemical coupling efficiency of ∼50% for the SecA motor,^52,53^ the hydrolysis of 3 ATP molecules would generate a mechanical work of 150 zJ (100 zJ per ATP × 50% efficiency × 3 molecules ATP). Although there are no single molecule force spectroscopy measurements of the forces generated by the SecA motor, it is thought to operate optimally over the force range of 5–11 pN, stalling at higher forces, which holds true for most protein translocating motors.^54,67^ This range coincides with the forces over which DsbA can assist protein folding, reaching a maximal effect at 9 pN (Fig. 3B). The refolding of a single protein L domain on the periplasmic side at 9 pN with a step size of 9.5 nm potentially generates a work of 85 zJ (9 pN × 9.5 nm), although the average amount of work performed is determined by the folding probability. This work of protein folding supplies a pulling force from the periplasm that can be harnessed to lower the amount of ATP consumed by SecA for translocation. Without DsbA in the periplasm, the folding of protein L can only generate an energy of 17.1 zJ (9 pN × 9.5 nm × 0.2 folding probability – see Fig. 3B). However, the presence of DsbA increases the work done by folding to 56 zJ (9 pN × 9.5 nm × 0.66 folding probability). Thus, the work of protein folding, assisted by the DsbA chaperone, can supply one-third of the energy needed for protein L translocation, lowering the ATP consumption to only 2 molecules per protein L polypeptide translocated. If the SecA motor has even lower efficiency, as suggested by certain studies demonstrating the requirement of 1000 ATP molecules per proOmpA transported, then the mechanical work done by DsbA is of even greater importance.^54^

There is emerging evidence to suggest that many chaperones transmit mechanical forces to their substrates during folding. The trigger factor increases the transmission force of the polypeptide through the ribosomal tunnel by assisting protein folding at higher forces.^26,55^ The results from the present study show how the chaperone activity of DsbA could assist the protein to translocate from the cytoplasmic end to the periplasmic end. Besides DsbA, other chaperones like HSP70 and BiP are known to assist the translocation process.^15,16^ It has even been demonstrated that high stability titin domains are transported into the lumen of the mitochondria with the help of mtHSP70.^17^ Another common chaperonin, GroEL, mechanically unfolds the misfolded proteins on its apical domain.^56^ Thus, a similar mechanical chaperone activity by all these chaperones indicates a broad mechanical role of chaperones which might be required in many biological processes such as the rescuing of stalled polypeptide chains in the translation process, accelerating protein folding in elastic tissues, or biasing the translocation of polypeptides from one cellular compartment to another.

As a factor involved in the maturation of a diverse set of bacterial exotoxins, adhesins, and secretion machinery, DsbA affords a single attractive target for attenuating a range of virulence phenotypes.^9,43^ The deletions of DsbA have been shown to alter the course of infection in animal models of *E. coli*^11^ and *P. mirabilis* urinary tract infection,^57^ and *F. tularensis* bacteremia,^58^ among others. Moreover, the loss of DsbA activity does not appreciably alter cell viability, lessening the selective drive for antibiotic resistance.^59^ To date, anti-DsbA therapeutic development has largely focused on inhibiting its oxidoreductase activity localized to the catalytic hydrophobic groove.^8,9,60^ Recent efforts have also explored potential small molecule and peptide binders of the non-catalytic groove on the opposite face, thought to be involved in protein–protein interactions.^10,61^ In this context, it is noteworthy that the PWATCDS peptide, which binds at the catalytic hydrophobic groove and inhibits the DsbA oxidase, also inhibits the mechanical foldase. In a related drug development program, a class of small molecules termed ‘pilicides’ are capable of preventing pilus formation by disrupting chaperone-mediated folding and assembly of the chaperone–usher system.^62–64^ In a similar vein, we propose that the redox-controlled DsbA chaperone activities open a novel avenue for anti-virulence antibiotic development.

## Methods

### Protein expression and purification

DsbA was purified from *E. coli* as described previously.^34^ The DsbA was used fresh after purification with storage at 4C and never after freezing. Oxidized DsbA was generated by incubating the enzyme in 10 mM oxidized glutathione overnight at 4 °C. Reduced DsbA was made by incubation with 100 μM TCEP overnight. The GSSG or TCEP was then removed by 3 × 1000 fold dilution into phosphate buffered saline (PBS) and centrifugal concentration. The oxidation state and concentration of the DsbA were determined with a DTNB assay and absorbance at 412 nm and 280 nm, respectively, using Ellman's test protocol described in the Goldbio protocol (https://www.goldbio.com/documents/2359/Ellmans+Test+Protocol.pdf) (ESI Fig. 8†). The eight-repeat protein L construct along with AviTag at the C-terminus and HaloTag enzyme at the N-terminus was expressed and purified as described before.^27^ I27^C32–C75^ was expressed and purified as previously described.^25,33^ PWATCDS peptide (99.3%) was purchased from Biomatik. During the peptide experiments with the protein L, we used 10 μM TCEP only with 50 μM reduced DsbA, not with oxidized DsbA. Paramagnetic Dynabeads (M-270) coated with streptavidin were purchased from Invitrogen. All the experiments were done using PBS as buffer.

### Magnetic tweezers instrumentation and coverslip preparation

Magnetic tweezers experiments were accomplished in our custom-made magnetic tweezers setups, with an inverted microscope (Zeiss Axiovert S100) mounted on a nanofocusing piezo actuator (P-725 PIFOC, Physik Instrumente), a magnet-positioning voice coil (LFA-2010, Equipment Solutions), and a high-speed camera (xiQ CMOS, Ximea GmbH). Fluid chambers are made of two cover slips sandwiched by two strips of parafilm. Both the top and bottom cover slips were washed by sonicating them separately for 30 min in 1% Hellmanex solution, 30 min in acetone, and 30 min in ethanol. The bottom slides are silanized by dipping them in a solution of (3-aminopropyl)-trimethoxysilane (Sigma-Aldrich) 0.1% v/v for 30 minutes in ethanol. After salinization, the bottom glass slides are then dried at 100 °C for an hour and then sandwiched with washed coverslips using laser-cut strips of parafilm, with a gentle heating at 85 °C for 1 minute. Then the chamber was functionalized with glutaraldehyde, nonmagnetic polystyrene beads (3 μm) and HaloTag amine ligand respectively as described previously.^27^ Recombinant octamer protein was tethered using HaloTag chemistry (N-terminal) and the other end of the protein was decorated with a single paramagnetic bead through biotin-streptavidin chemistry (AviTag, Avidity Technology). The position of the permanent magnets was controlled with a linear voice-coil with a speed of ∼0.7 m s^−1^ and 150 nm position resolution. The force on the protein is calibrated by the procedure mentioned by Popa *et al.*^27^ Experiments with DsbA are performed after >15 minutes of equilibration with protein L in the fluid chamber. Similarly, PWATCDS peptide is incubated for >30 minutes with DsbA before adding to the fluid chamber.

### Data analysis

Only proteins showing eight unfolding events in the high force (45 pN) fingerprint pulse are considered for analysis. After unfolding the protein, the force is relaxed during the “quench” pulse to achieve an equilibrium between the folding and unfolding states to calculate the folding probability. We measure this by calculating the probability of finding the system in a particular state, considering that *t*_*i*_ is the total time the system spends in state *i* (where *i* equals the number of folded domains, *i* = 0, 1, …, *N*) and *t*_t_ the total time of experiment, 
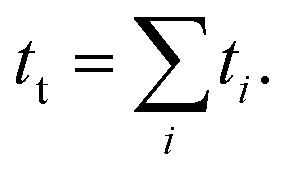
 Then, the folding probability is 
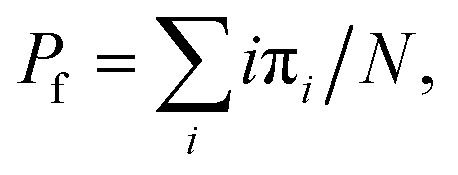
, where π_*i*_ = *t*_*i*_/*t*_t_. This was the same metric used in previous magnetic tweezers based chaperone assays.^26^

## Data availability

All data supporting the results and conclusions are available within this paper and the ESI.

## Author contributions

S. H., E. C. E., and D. J. E. designed the project, S. H., E. C. E., D. C., and S. C. performed the experiments and analyzed the data, and S. H., D. J. E., and E. C. E. wrote the paper.

## Conflicts of interest

The authors declare no conflict of interest.

## Supplementary Material

SC-012-D1SC03048E-s001
