# Supplementary material for: DsbA is a redox-switchable mechanical chaperone
Source: Chem Sci. 2021 Jul 19;12(33):11109–20. doi: 10.1039/d1sc03048e (PMC8386657; doi:10.1039/d1sc03048e)
Supplement: SC-012-D1SC03048E-s001 [file SC-012-D1SC03048E-s001.pdf]

Electronic Supplementary Material (ESI) for Chemical Science.
This journal is © The Royal Society of Chemistry 2021

Supplementary Information

DsbA is a reox-switchable Mechanical Chaperone

Edward C. Eckels[2, 3, †, *], Deep Chaudhuri[1, †], Soham Chakraborty[1],

Daniel J. Echelman[2], Shubhasis Haldar [1, *]

[1]Department of Biological Sciences, Ashoka University, Sonepat, Haryana 131029

[2]Department of Biological Sciences, Columbia University, New York, NY 10027

[3]Department of Internal Medicine, Columbia University Medical Center, New York NY 10032

[†] Contributed equally to the work

* To whom correspondence may be addressed: shubhasis.haldar@ashoka.edu.in or ece7001@nyp.org

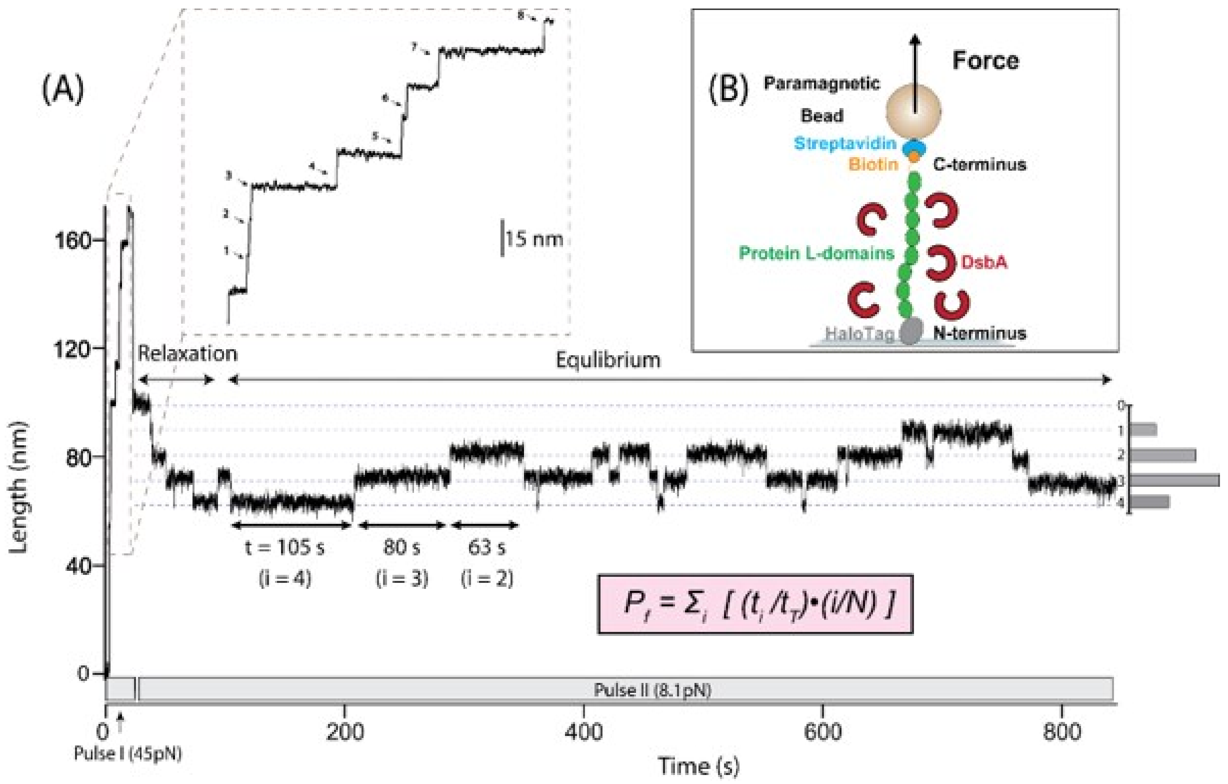

Supplementary Figure 1. Folding probability under force measured by magnetic tweezers (A) Representative magnetic tweezers demonstrating unfolding and refolding transitions of an eight-repeat (*N*=8) construct of the protein L domain: The protein is first unfolded at a constant force of 45 pN (Pulse I) resulting in eight consecutive unfolding (upwards-step) transitions of 15 nm each (see inset for magnified). The force is then quenched to 8.1 pN (Pulse II) resulting in entropic recoil of the protein followed by relaxation to an equilibrium between folding (downwards-step) and unfolding transitions. The state *i* of the protein at any point during the recording is equal to the number of folded domains. The total residence time (t_i) of each folded state during the equilibrium phase is shown by the gray histogram on the right and the sum of all residence times (t_T) is equal to the duration of the equilibrium phase. After calculating the states *i* and the residence times t_i the folding probability at a force of 8.1 pN is calculated according the presented equation (pink inset). **(B) Schematic of the magnetic tweezers experiment:** One end of octamer of protein L is attached to the glass coverslip via HaloTag covalent chemistry and the other end is tethered to a paramagnetic bead via biotin-streptavidin binding. A precise pulling force is applied by positioning a pair of permanent magnets above the tethered paramagnetic bead with sub-micron resolution. DsbA (red curls) can be washed into or out of the flow cell during the course of an experiment.

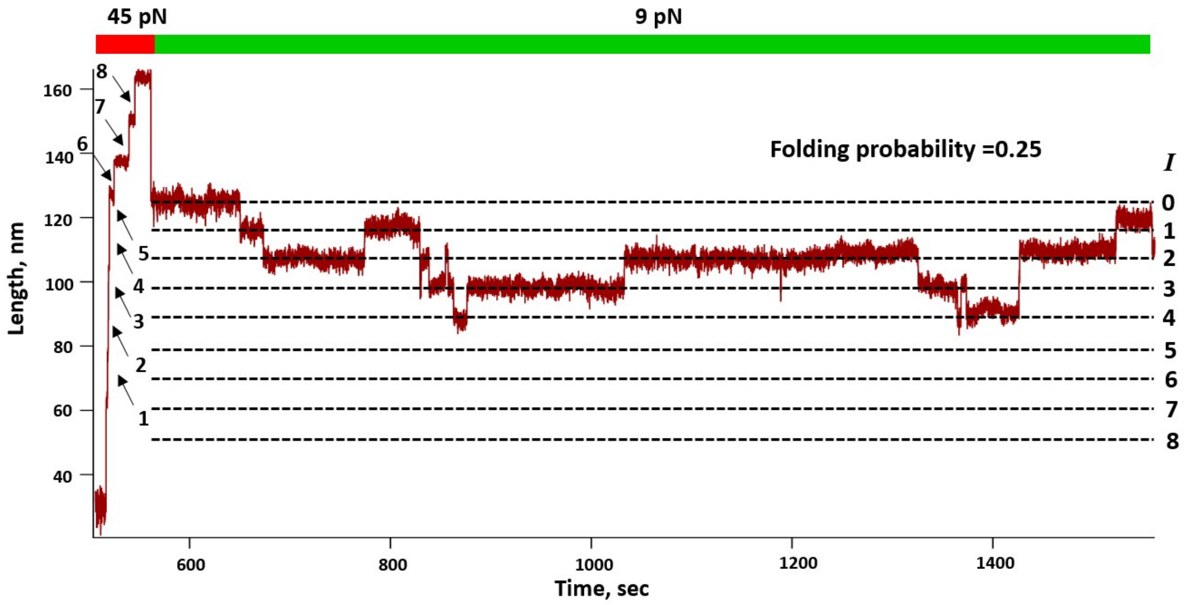

Supplementary Figure 2: Representative trace of protein L in the presence of 3μM reduced DsbA: In the first pulse, protein L is completely unfolded at 45 pN, where it shows eight unfolding (finger print) steps. Then in the next pulse, it is quenched to 9 pN, where proteins stay in an equilibrium state and showing folding-unfolding dynamics. The calculated folding probability at 9 pN is 0.25.

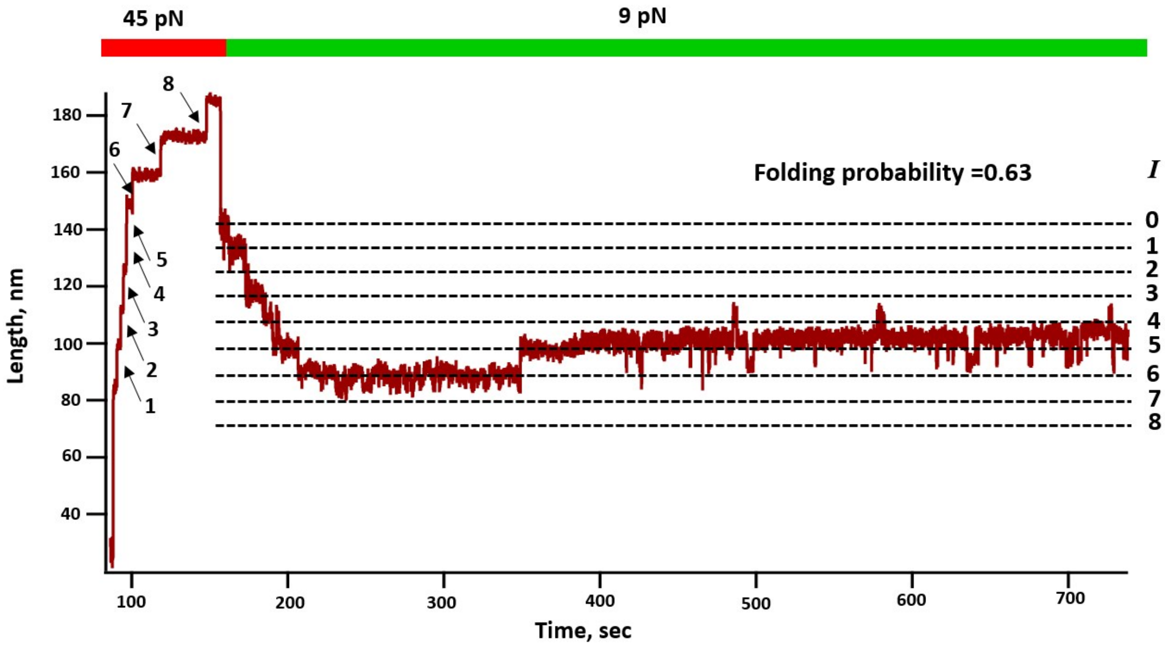

Supplementary Figure 3: Representative trace of protein L in the presence of 50 µM reduced DsbA: Similar to supplementary Figure 1, in the first pulse protein L is unfolded at 45pN and then quenched at 9 pN. In presence of 50 µM reduced DsbA, folding probability protein L increased to 0.63 at 9 pN.

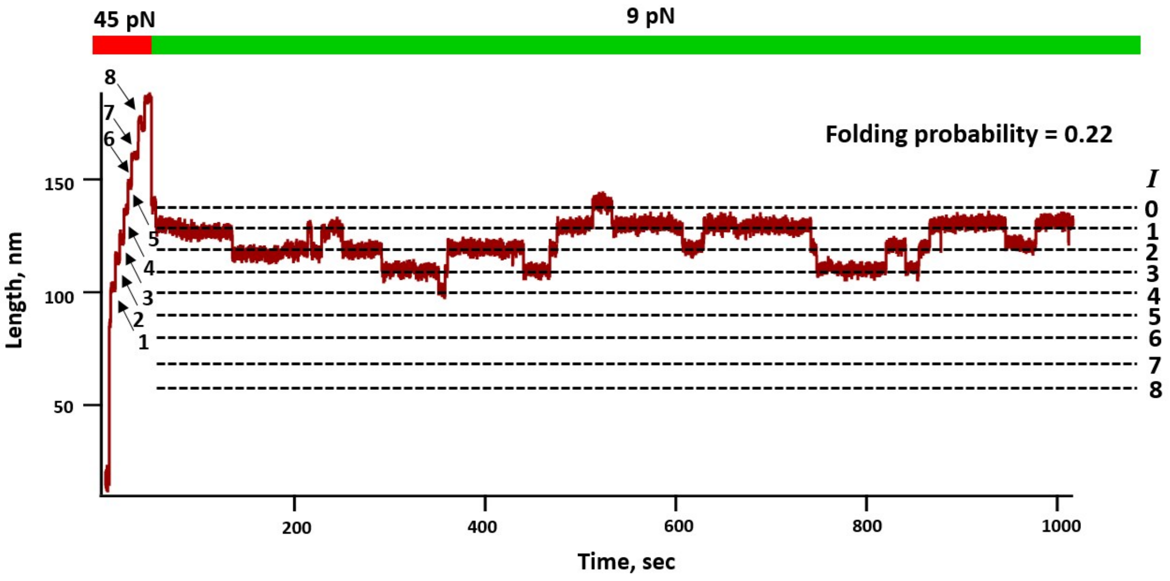

Supplementary Figure 4: Representative trace of protein L in presence of 3 µM DsbA and 100 µM PWATCDS peptide: Similar to first two figures, protein L was unfolded and folded in the presence of 3 µM oxidised DsbA and 100 µM PWATCDS peptide. In presence of the peptide, the folding probability of Protein L decreases to 0.22 at 9 pN.

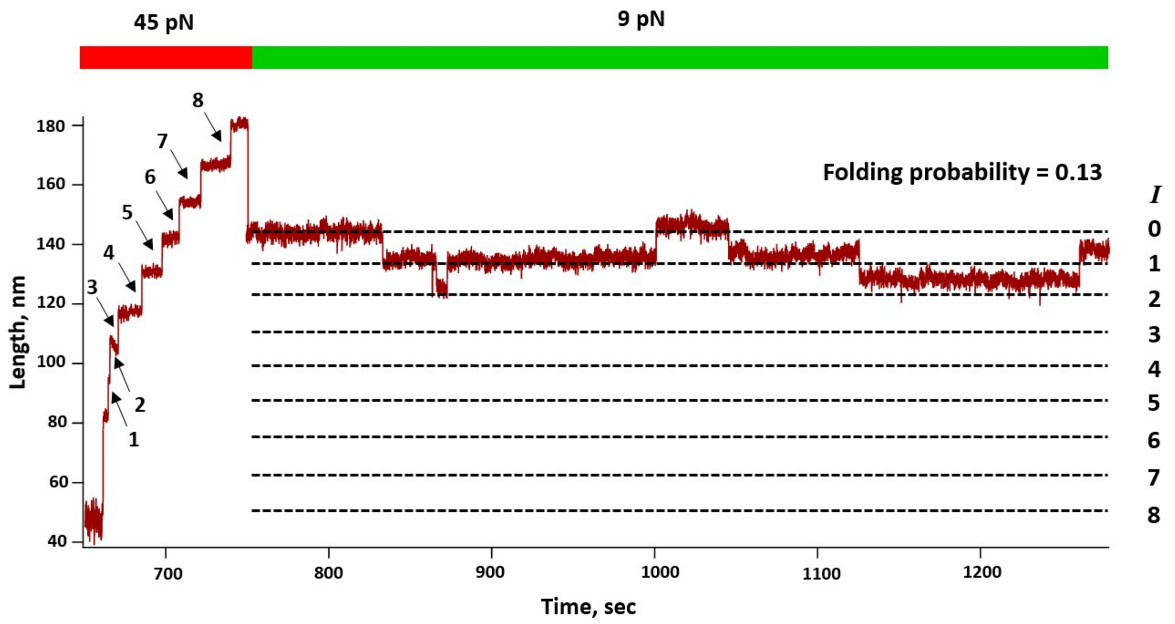

Supplementary Figure 5: Representative trace of protein L in the presence of 50 µM reduced DsbA and 100 µM peptide: Similar to previous 3 figures, in the first pulse the protein unfolds fully at 45 pN and then in the second pulse, it refolds to 9 pN force. In presence of peptide protein L shows a folding probability of 0.13.

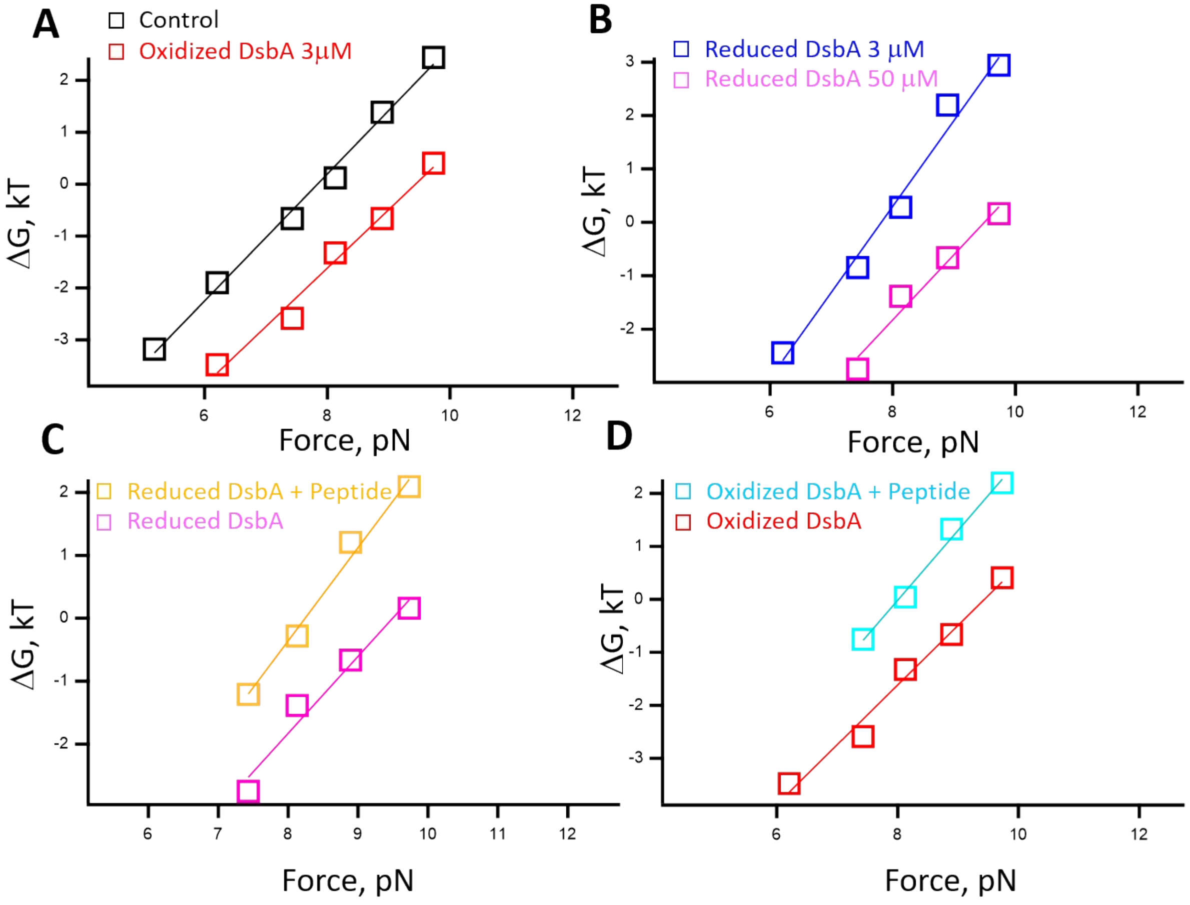

Supplementary Figure 6: ΔG folding is calculated from the folding probability values using the protocol described in Chen et al., (JACS, 2015, 137 (10), 3540-3546). The ΔG folding is measure for protein L in the presence of 3 μM oxidized DsbA (red), 3 μM reduced DsbA (blue), 50 μM reduced DsbA (pink), 3 μM oxidized DsbA and peptide (cyan), 50 μM reduced DsbA and peptide (yellow) and control (black).

MFPT Calculations

Mean first passage times (MFPT) were calculated by averaging the trajectory durations for n > 5 molecules. To calculate the folding MFPT, the protein construct was first fully unfolded, the force was relaxed and the time required to achieve folding of all eight domains simultaneously was recorded. This was performed for several different folding forces in the range of 4-8 pN and was then fit with a single exponential equation of the form $MFPT(F) = A\exp -F/\varphi$, and the fit parameters are recorded below:

	A (ms)	*φ (pN)*
DsbA	0.784	-0.632
Control	0.269	-0.505

Supplementary Table 1: *MFPT refolding fit parameters for DsbA and control conditions with protein L*

Likewise, for MFPT of unfolding, the protein construct is first allowed to fold completely at a low force before it is probed at a high force of 30-50 pN. The time required to pass to the state with all eight domains unfolded simultaneously is recorded and averaged for several molecules. The same equation is used to fit the MFPT and the parameters are recorded below:

	A (s)	*φ (pN)*
DsbA	2230	9.52
Control	3267	8.13

Supplementary Table 2: *MFPT unfolding fit parameters for DsbA and control conditions with protein L*

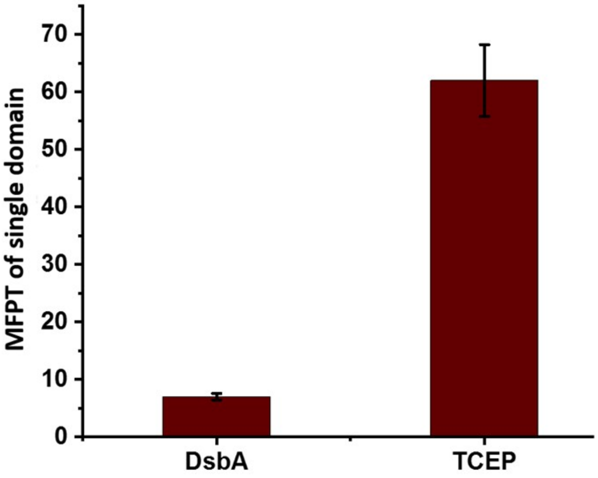

Supplementary Figure 7: MFPT of single I27 domain: In the presence of TCEP, the refolding time of a single I27 domain is 62±6.25s, which reduces to 7±0.57s in the presence of DsbA. This result shows that DsbA increases the rate of folding.

Determination of oxidation state by DTNB assay

We checked the percentage of the oxidized and reduced DsbA using Ellman's test protocol (https://www.goldbio.com/documents/2359/Ellmans+Test+Protocol.pdf). After purification, we oxidized the DsbA by incubating with 0.3% H2O2 and reduced it by incubating with 100 μM TCEP for overnight at 4ºC.

The oxidized and reduced fractions are then purified by the size exclusion chromatography. Using these samples, we performed the magnetic tweezers experiments and DTNB assay. DTNB assays are used to measure the percentage of oxidized and reduced DsbA. Our results shows for oxidized DsbA, more than 99% DsbA molecule is oxidized; whereas for the reduced one, 98.75% DsbA molecule are reduced.

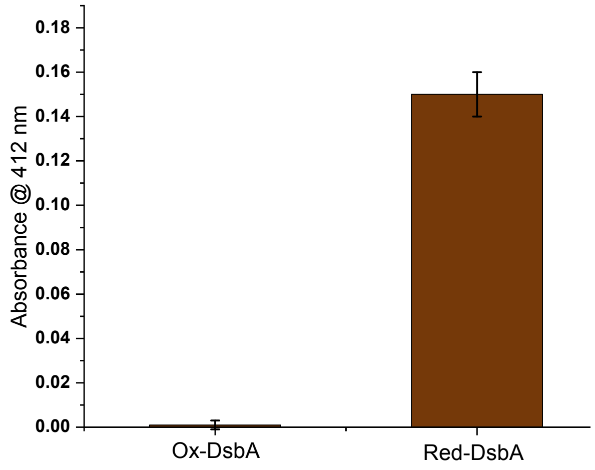

Supporting Figure 8: DTNB assay to determine the oxidation state: For oxidized DsbA, the absorbance at 412 nm is almost 0, indicating no free thiols or the presence of any reduced DsbA which concludes that more than 99% DsbA is oxidized by H_2O_2. In case of reduced DsbA, 5.58 µM reduced DsbA was used in DTNB assay. We measured the absorbance 0.15 at 412 nm, which shows 5.51 µM DsbA is reduced. This result shows our reduced sample is 98.75% reduced.

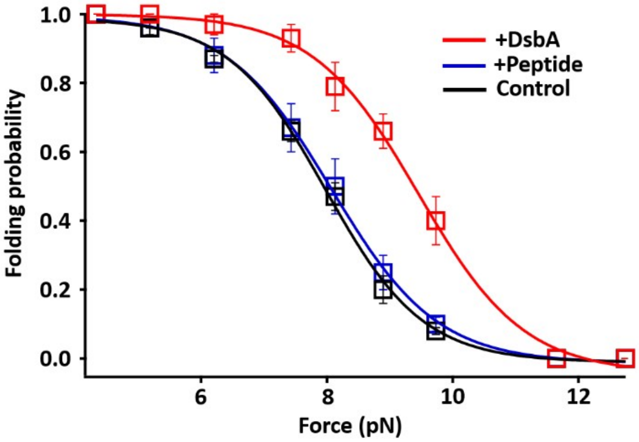

Supporting Figure 9: Effect of peptide (PWATCDS) on protein L refolding: Folding probabilities of protein L, only in the presence of DsbA (red) and peptide (blue) are plotted against the force. The peptide does not interact with the protein L and thus, does not shift the folding probability and it overlaps with the control (black), whereas the DsbA has been observed to increase the folding probability. Each data point is calculated using > 2500s and over three molecules per force. Error bars represent standard error of the mean.
